# Identification of microRNAs as biomarkers for triage of cervical intraepithelial neoplasia in cervical scrape samples from high-risk HPV-positive women

**DOI:** 10.1038/s41416-026-03521-y

**Published:** 2026-08-04

**Authors:** Lidy Vannessa Mejia Guarnizo, Clara Esperanza Trujillo Gama, Liliana López kleine, Carlos Alberto Orozco Castaño, Sebastián Prada Padilla, Josefa Antonia Rodríguez García, María Mercedes Bravo Hernandez

**Affiliations:** 1 Grupo de Investigación en Biología del Cáncer—Instituto Nacional de Cancerología, Bogotá, Colombia; 2 Maestría en Ciencias Microbiología, Universidad Nacional de Colombia—sede Bogotá, Bogotá, Colombia; 3 Departamento de Estadística, Facultad de Ciencias, Universidad Nacional de Colombia– sede Bogotá, Bogotá, Colombia

**Keywords:** Tumour biomarkers, Cervical cancer, Human papilloma virus, miRNAs, Diagnostic markers

## Abstract

**Background:**

Cervical cancer (CC) is the fourth leading cause of cancer death in women worldwide. High-risk human papillomavirus (HR-HPV) testing is highly sensitive but lacks specificity for identifying women at risk of progression. Reliable biomarkers are urgently needed to identify HR-HPV-positive women most likely to develop cervical cancer. Aberrant microRNA (miRNA) expression contributes to HPV-driven carcinogenesis and represents a promising diagnostic tool.

**Methods:**

Cervical scrape samples from HR-HPV-positive women were histologically classified as low-grade (≤CIN1) or high-grade (CIN2/3). Total RNA was extracted and small RNA sequencing performed to identify differentially expressed miRNAs. Bioinformatic analyses included clustering, ROC curves, logistic regression, and functional enrichment. Selected candidates were validated byRT-qPCR in independent samples.

**Results:**

Nineteen dysregulated miRNAs distinguished low- from high-grade lesions, six linked to viral and cancer pathways. Three (hsa-miR-501-3p, hsa-miR-1271-5p, hsa-miR-9-5p) were validated by RT-qPCR and showed strong discriminatory capacity. hsa-miR-501-3p reached high diagnostic accuracy (AUC = 0.88), which improved to 0.94 when combined with the others. The validated signature achieved high sensitivity and specificity for detecting high-grade lesions.

**Conclusion:**

This pioneering study in Colombia using cervical scrapes demonstrates that miRNAs are potential biomarkers for high-grade cervical lesions and that this clinically relevant, minimally invasive sample routinely collected in screening supports their use.

## Introduction

CC remains a major global public health challenge, especially in low- and middle-income countries where approximately 85% of global cervical cancer cases and deaths occur [1]. In Colombia, the incidence rate was estimated at 13.7 per 100,000 women, ranking third after breast and colorectal cancer [2]. While high-risk human papillomavirus (HR-HPV) testing has improved the sensitivity of cervical cytology-based screening programs, its limited specificity leads to over-referral for colposcopy of HPV-positive women who may never develop high-grade lesions, with implications for costs and patient well-being. Despite its widespread use, Cervical cytology has limited sensitivity as it is based in subjective morphological criteria [3], and HR-HPV DNA genotyping suffer from subjectivity because it does not fully capture the risk associated with other oncogenic types or accumulated host molecular alterations [4–6], highlighting the need for molecular biomarkers more objective, reproducible, and suitable for high-throughput platforms, to better classify HPV-positive women according to their risk of progression.

MicroRNAs (miRNAs), small non-coding RNAs that regulate gene expression post-transcriptionally [7–9], by binding their target genes, triggering silencing mechanisms such as translational repression, deadenylation, decapping, and 5′-to-3′ mRNA degradation, have emerged as promising candidates as biomarkers to improve risk stratification for HR-HPV positive women [10]. miRNA dysregulation has been linked to cervical carcinogenesis, where miRNAs may function as oncogenes (oncomiRs) or tumor suppressors [7]. In the context of high-risk HPV infection viral oncoproteins such as E6 and E7 disrupt key tumor suppressor pathways, including p53 and pRb, leading to broader alterations in host gene expression networks that also affect miRNA expression profiles [8]. As biomarkers, miRNAs are promisors because they are highly stable in body fluids, resistant to RNAase degradation, and can be detected in cervical scrapes samples, and offer a minimally invasive tool for early diagnosis [11].

Although many studies have evaluated miRNA profiles in cervical tissue or biopsies, [7, 9, 12, 13], their utility in cervical scrapes samples, routinely used in CC screening, remains underexplored. Cervical scrapes are an accessible, minimally invasive, and widely available source of biological material, ideal for molecular analysis and for the implementation of triage strategies without requiring additional or invasive procedures.

In this study, we analyzed the miRNA expression profiles in cervical scrapes samples from HR-HPV-positive women with low-grade and high-grade lesions, aiming to identify miRNAs with potential utility as biomarkers for improved triage and early detection of precancerous cervical lesions. Our approach seeks to provide evidence on the feasibility of integrating miRNA analysis into the current screening and triage framework, advancing toward more precise and objective strategies for early detection of high-grade cervical lesions.

## Methods

### Study design, population, and sample collection

**Fig. 1:**
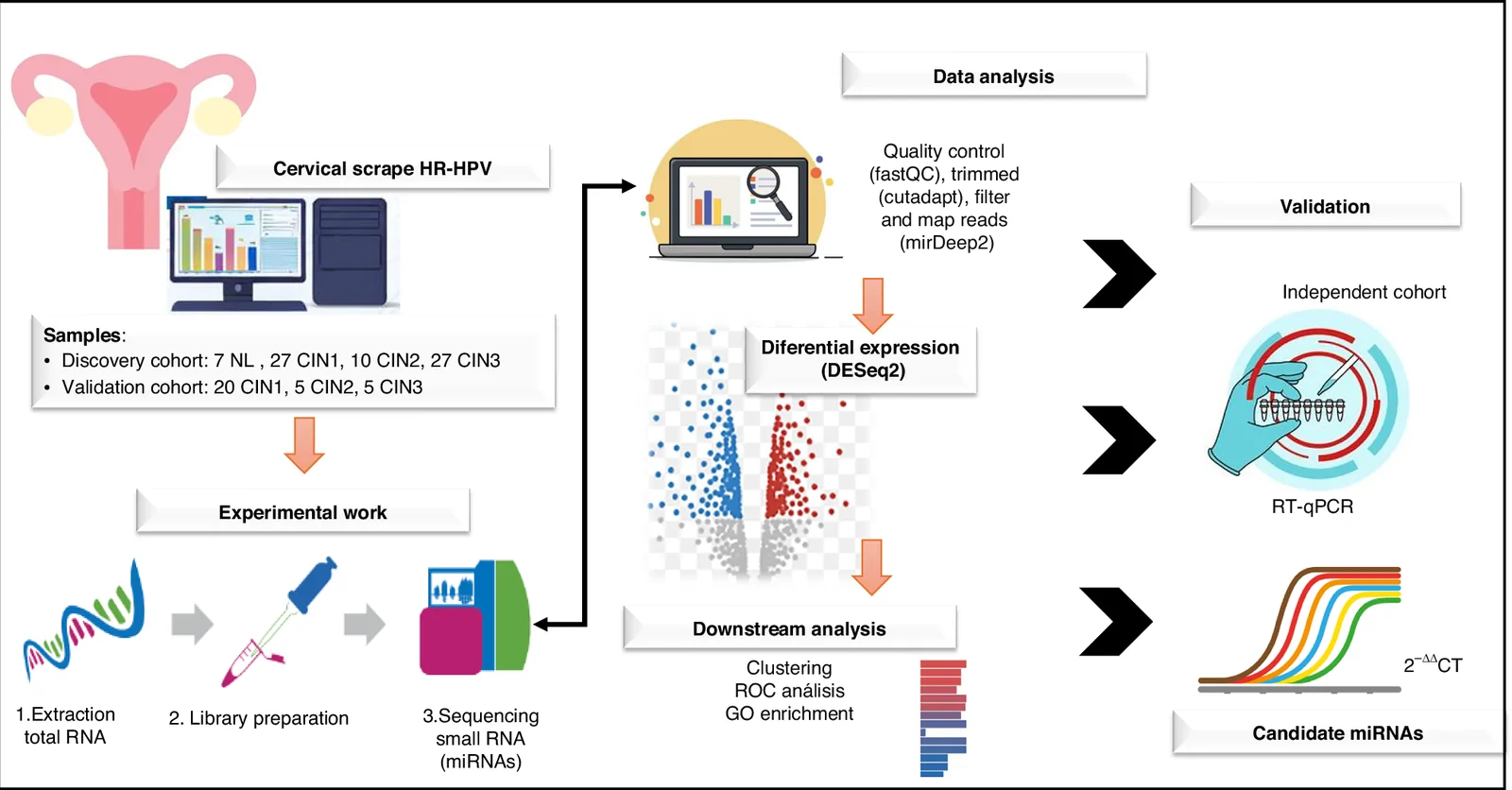
Schematic representation of the experimental and bioinformatic workflow.

This cross-sectional study analyzed HR-HPV-positive cervical samples stratified by histology (≤CIN1 vs. CIN2/3), to identify differentially expressed miRNAs as potential triage biomarkers. (Fig. 1). Samples were selected from a previously characterized cohort of 315 women recruited at the Instituto Nacional de Cancerología (INC) between 2010 and 2012, and inclusion/exclusion criterio were applied to ensure homogeneity. The study included women positive for high-risk HPV with histologically confirmed cervical lesions (≤CIN1 or CIN2/3). Individuals with conditions that could affect cervical status or miRNA expression, such as pregnancy or concurrent infections, were excluded.

No individual matching was performed between lesion groups. The discovery set included 71 samples (34 ≤ CIN1 and 37 CIN2/3) and 30 independent samples (20 CIN1 and 10 CIN2/3) selected under the same criteria as the discovery set, were used for qPCR validation. Sample size was calculated using the ssizeRNA R package (power = 80%, FC = 2, a = 0.05). Supplementary Tables S1 and  S2 show the baseline sociodemographic and clinical characteristics of patients in the discovery and validation sets, respectively, which were compared between lesion-grade groups to assess potential imbalances and reduce selection bias.

Cervical samples were collected prior to colposcopic evaluation using standardized cervical brushing. A trained physician obtained each sample with a sterile cytobrush, rotating 360 in the cervical transformation zone to maximize cell yield. Brushes were immediately placed into 500 μL of RNA lysis buffer (SV Total RNA Isolation System, Promega) and stored at −80 °C until RNA extraction [14]. Clinical, cytological, histological, and HPV typing data were collected for all participants.

This study was approved by the ethics committee of the INC and adhered to the ethical standards set forth by the Helsinki Declaration and Colombian legislation. A designated research team member conducted the informed consent process, addressed any participant questions or concerns, and all included participants signed an informed consent form.

### RNA extraction, library preparation and processing data sequence

Total RNA was extracted from cervical scrape samples using the Quick-RNA™ Miniprep Kit (ZYMO Research), which allows efficient recovery of small RNAs. RNA concentration and quality was assessed with NanoDrop, Qubit® Fluorometer and the Agilent 2100 Bioanalyzer to obtain RNA Integrity Number (RIN) values. RIN values were recorded for all samples. All samples proceeded to small RNA library preparation and sequencing. Individual RIN values are provided in Supplementary Table S3.

Small RNA libraries were prepared with the Illumina TruSeq Small RNA Library Prep Kit, following the manufacturer’s instructions. This included adapter ligation, reverse transcription, and PCR amplification. Library size distribution was evaluated with the Agilent High Sensitivity DNA 1000 chip, and concentrations were quantified with the Qubit™ dsDNA HS Assay kit. Equimolar pools (4 nM) of up to four samples were sequenced on the Illumina MiSeq platform (50-base single-end reads).

Raw reads were quality-filtered (Phred score ≥30; length 15–27 nt) with FastQC, adapter trimmed with Cutadapt [15] and aligned to the reference transcriptome using miRDeep2 [16]. The mapper.pl module was used to align sequences to the GRCh38 reference genome and mature miRNAs were quantified using the quantifier.pl module with annotations from miRBase v21. Post-sequencing sample-level quality control was performed through exploratory data analysis, including count distribution assessment and inter-sample correlation analyses, to identify potential outliers prior to differential expression analysis.

### Differential expression analysis

Raw miRNA counts were normalized and analyzed for differential expression using DeSeq2 [17]. Differential expression analysis was performed by comparing miRNA expression levels between the ≤CIN1 and CIN2/3 groups using the Wald test.

miRNAs with low counts, or samples with atypical count distributions and low inter-sample correlation (Pearson’s r < 0.80) were excluded based on exploratory quality-control plots. *P* values were corrected for multiple comparisons using the Benjamini-Hochberg method to obtain false discovery rates (FDR). miRNAs with FDR < 0.05 and |log₂FC | > 1 were considered significantly differentially expressed. All Analyses were performed in Rstudio (v4.3.2) following the DESeq2 workflow.

### GEO dataset reanalysis

To validate our findings, we reanalyzed two public datasets (GSE104758 and GSE167858) using the same DESeq2 pipeline. Only datasets from HPV-positive cervical samples were included and overlapping differentially expressed miRNAs were identified to assess reproducibility across independent cohorts.

### Target prediction and functional enrichment analysis

Experimentally validated miRNA–mRNA interactions were performed using the multiMiR package in RStudio (v4.3.2) [18], limited to human targets from miRecords, miRTarBase, and TarBase databases. Functional enrichment of target genes was carried out using the clusterProfiler package [19], applying enrichGO and enrichKEGG for Gene Ontology (GO) terms classified into Biological Process (BP), Molecular Function (MF), and Cellular Component (CC), and KEGG pathways, respectively. Significance was defined as adjusted p < 0.05 (FDR). A miRNA–mRNA interaction network was visualized based on functional relevant targets related to HPV-mediated carcinogenesis using igraph and ggraph packages.

### Clustering

Unsupervised clustering was applied as an exploratory strategy to evaluate the ability of differentially expressed miRNAs to distinguish samples based on clinical diagnosis (≤CIN1 vs. CIN2/3). Normalized expression data and transformed counts were subjected to principal component análisis (PCA). Clustering was then performed using k-means (k = 2), assuming two main clinical categories. The analysis was conducted using the ade4, factoextra, and corrplot packages in RStudio.

This approach allowed the visualization of intrinsic grouping patterns and the identification of miRNAs with the highest contribution to group separation (based on their loading values on principal components). Additionally, correlation analysis was used to filter redundant signals and refine the list of candidate miRNAs.

### Receiver operating characteristic curves (ROC)

ROC curves were performed using SPSS Statistics software version 25. Normalized miRNA expression counts were used as input data. Groups of interest were defined as SL/NIC1 versus NIC2/3. The median expression of each miRNA was used to determine its score. A score of 1 was assigned if the expression was above, and 0 if it was below the median.

### Logistic regression

A univariate logistic regression was performed, to identify miRNAs associated with high-grade preneoplastic lesions (CIN2/3) analyzing the relationship between miRNA levels, socioeconomic variables, and the presence of high-grade lesions (1) versus the absence or presence of low-grade lesions (0). Each candidate miRNA was evaluated individually, selecting those with a *p* value ≤ 0.05, indicating a statistically significant association. In addition, Odds Ratios (OR) and their 95% confidence intervals (95% CI) were calculated. The analysis was performed with SPSS Statistics software version 25.

### Validation of miRNA candidates by RT-qPCR

miRNAs selected for RT-qPCR validation were chosen based on their strong differential expression and recurrent identification across complementary analyses (clustering, ROC curve evaluation, and logistic regression). In addition, hsa-miR-99a-5p was included due to its marked differential expression.

Selected miRNAs were validated in 30 independent samples. Total RNA, including small RNAs, was extracted with the Quick-RNA™ Miniprep Kit (ZYMO Research). Reverse transcription was performed using the TaqMan™ MicroRNA Reverse Transcription Kit (Thermo Fisher Scientific) with pooled primers, using 25 ng RNA as template. qPCR was carried out on the QuantStudio 3 System with TaqMan® MicroRNA Assays, using hsa-miR-423-3p as endogenous control. Only samples with Ct ≤ 32 were analyzed.

### Statistical analysis of RT-qPCR data

Relative expression levels were calculated using the ΔΔCt method [20]. Normality tests of continuous numerical variables were performed using the Shapiro-Wilk test. To compare medians between CIN1 and CIN2/3 groups, the Mann–Whitney U test was used, and for multiple comparisons (CIN1, CIN2, CIN3) the Kruskal-Wallis test followed by the Dunn test were applied. Statistical and graphical analyses were performed using GraphPad Prism. ROC curve analyses were used to assess diagnostic performance separating CIN2/3. A multivariate logistic regression model was fitted to generate a combined score estimating the probability of a sample belonging to the CIN2/3 group. The area under the curve (AUC) and its 95% confidence interval were calculated to measure the accuracy of the marker. Optimal cutoff points were determined using Youden and MaxSp methods.

## Results

### Discovery phase

#### Clinicopathological features of the study sample

The study included two independent cohorts: a discovery set of 71 HPV-positive women (34 with ≤CIN1 and 37 with CIN2/3) and a validation set of 30 women (20 ≤ CIN1 and 10 CIN2/3). Clinical and sociodemographic variables including age, education level, occupation, parity, contraceptive use, and smoking status were assessed to characterize the study population and explore potential associations with lesion grade. No statistically significant differences were observed between the lesion grade groups in either cohort. Although a higher proportion of women aged over 30 and those with three or more births was noted in the CIN2/3 group of the discovery cohort, these trends did not reach statistical significance.

Similarly, no significant associations were found for other variables such as contraceptive use, education level, smoking status, or occupation. These results suggest that the cohort had comparable baseline characteristics (Supplementary Tables S1 and S2).

#### Dysregulated miRNA profile in high- and low-grade cervical lesions

The discovery set included cervical scrape samples from 71 HPV-positive women: 37 with high grade lesions (CIN2/3) and 34 with low-grade lesions or no histological abnormalities (≤CIN1). For clarity, samples with no lesion were labeled as NL (No Lesion). Small RNA sequencing generated an average of 3.9 million raw reads per sample, with ~3.3 million reads remaining after preprocessing. Of these, approximately 35.7% mapped to known miRNAs (miRBase v21). Sequencing performance metrics, including read yield and mapping proportion, were comparable across samples despite variability in RIN values, indicating that RNA integrity differences did not prevent successful small RNA profiling.

**Fig. 2:**
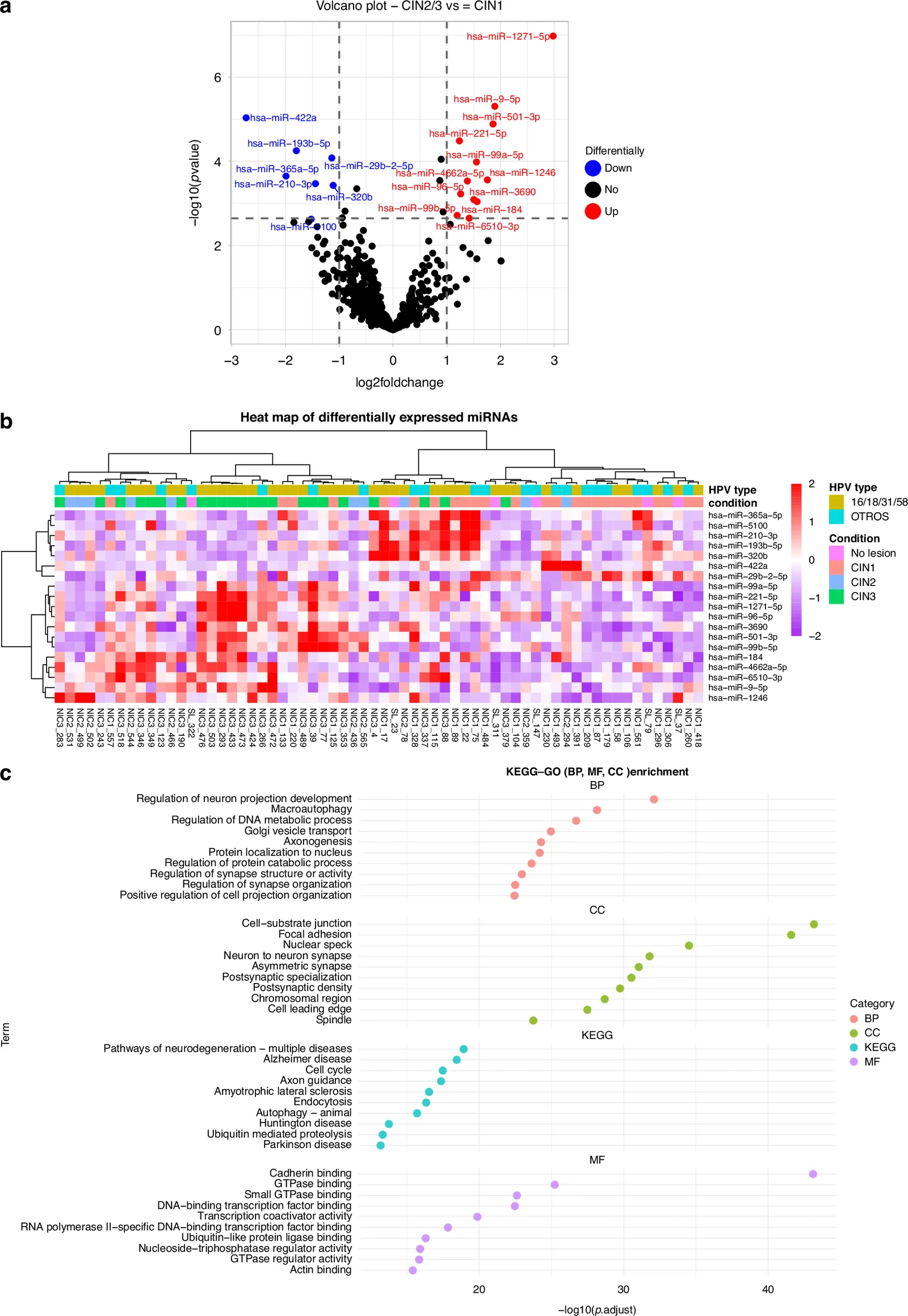
Differentially expressed miRNAs associated with cervical lesion grade. **a** Volcano plot showing miRNAs significantly upregulated (red) or downregulated (blue) in high-grade lesions (CIN2/3) compared to low-grade lesions (≤CIN1). **b** Heatmap of the 19 differentially expressed microRNAs between CIN2/3 (*n* = 31) and NL/CIN1 (*n* = 31) lesions. Rlog-transformed counts were row-scaled and clustered using correlation distance and the Ward. method. Red indicates higher relative expression; purple indicates lower expression. **c** Top enriched Gene Ontology (GO) terms identified from the target genes of differentially expressed miRNAs in cervical preneoplastic lesions, categorized into Biological Process (BP), Molecular Function (MF), and Cellular Component (CC).

Following quality control, 7 samples were excluded, resulting in a final dataset of 64 samples (33 CIN2/3 and 31 ≤ CIN1). After filtering out low-abundant transcripts, 717 miRNAs were retained for downstream analysis. Differential expression analysis between CIN2/3 and ≤CIN1 groups identified 19 significantly dysregulated miRNAs (adjusted *p* ≤ 0.05; logFC >1), with 12 upregulated and 7 downregulated in the high-grade group (Fig. 2a, Table 1). Additional pairwise comparisons (NL vs. CIN1, NL vs. CIN2/3, CIN1 vs. CIN2/3) supported the consistency of these findings (Supplementary Fig. S1).

**Table 1:** Table displaying the 19 most significantly differentially expressed miRNAs.

miRNA	baseMean	FC	log2FC	*p* value	padj	Expressed
***hsa-miR-1271-5p***	8.94	7.87	2.97	1.05E–07	5.50E–05	UP
hsa-miR-9-5p*	60.34	3.71	1.89	4.92E–06	0.0012	UP
***hsa-miR-501-3p***	167.42	3.63	1.85	1.29E–05	0.0016	UP
***hsa-miR-221-5p***	50.62	2.35	1.23	3.26E–05	0.0034	UP
hsa-miR-99a-5p*	491.60	2.93	1.55	0.00010	0.0060	UP
hsa-miR-1246*	11.06	3.37	1.75	0.00027	0.011	UP
***hsa-miR-4662a-5p***	3.67	2.60	1.38	0.00029	0.011	UP
***hsa-miR-96-5p***	38.60	2.39	1.25	0.00059	0.018	UP
*hsa-miR-3690*	16.53	2.83	1.50	0.00080	0.023	UP
hsa-miR-184*	96.24	2.95	1.56	0.00090	0.024	UP
*hsa-miR-99b-5p*	3125.91	2.28	1.19	0.0019	0.045	UP
*hsa-miR-6510-3p*	9.42	2.67	1.41	0.0022	0.048	UP
***hsa-miR-422a***	5.31	0.15	-2.73	9.23E–06	0.0016	DOWN
***hsa-miR-193b-5p***	13.87	0.28	-1.79	5.59E–05	0.0048	DOWN
***hsa-miR-29b-2-5p***	3.55	0.45	-1.13	8.30E–05	0.0058	DOWN
***hsa-miR-365a-5p***	4.60	0.25	-1.99	0.00022	0.011	DOWN
***hsa-miR-210-3p***	1189.88	0.36	-1.44	0.00033	0.012	DOWN
***hsa-miR-320b***	430.58	0.46	-1.11	0.00037	0.012	DOWN
*hsa-miR-5100*	90.067	0.34	-1.52	0.0023	0.049	DOWN

*miRNAs shared with additional internal comparisons are shown in bold. and those replicated in external public datasets are indicated by an asterisk (*).

identified by miRNA-seq. with adjusted *p* values and fold change. in the comparison between CIN2/3 and the reference group ( ≤ CIN1).

Hierarchical clustering of the 19 differentially expressed miRNAs revealed two principal sample clusters (Fig. 2b). One cluster was primary composed by CIN2/3 cases, while the other included most ≤CIN1 cases. Some overlap was observed, particularly within CIN2 cases, reflecting the known biological heterogeneity of this category. Notably, elevated expression of specific miRNAs was more frequent in CIN2/3 lesions, whereas lower expression levels were characteristic of NL and CIN1 samples, supporting their potential utility for discriminating lesion severity.

#### Validation in independent public datasets

To strengthen the robustness of our findings and assess the external reproducibility of the differentially expressed miRNAs identified in our cohort, we analyzed two independent cervical lesion datasets from the Gene Expression Omnibus (GSE104758 and GSE167858), both with comparable experimental designs and lesion classification criteria. Using the same bioinformatic pipeline, we identified a subset of miRNAs including hsa-miR-1246, hsa-miR-9-5p, hsa-miR-184, and hsa-miR-99a-5p that exhibited concordant expression patterns with those observed in our cohort (Supplementary Fig. S2). These results reinforce the potential of these miRNAs as robust biomarkers for high-grade cervical lesions in HPV-positive women and support their consistency across independent datasets.

#### Functional enrichment analysis

To gain insight into the biological functions potentially regulated by the differentially expressed miRNAs, we performed a functional enrichment analysis based on their predicted target genes. Targets were retrieved from experimentally validated interactions and high-confidence computational predictions. The analysis revealed a significant overrepresentation of pathways associated with carcinogenic processes, including cell adhesion, chromatin remodeling, and intercellular signaling.

Among the most enriched terms were focal adhesion, histone modification, and cell cycle regulation key pathways implicated in the progression of HPV-driven cervical lesions (Fig. 2c). A complete list of enriched terms, along with the corresponding genes and associated miRNAs, is provided in Supplementary File 1.

#### Exploring the capacity of miRNAs to distinguish between low- and high-grade cervical lesions

Although numerous miRNAs were found to be differentially expressed and validated in independent datasets, differential expression alone does not necessarily imply discriminatory performance at the individual sample level. Therefore, we evaluated whether these miRNAs were able to distinguish between low- and high-grade cervical lesions using complementary analytical approaches.

An unsupervised clustering analysis using the K-means algorithm was applied to assess whether miRNA expression profiles could stratify samples according to histological classification (≤CIN1 vs. CIN2/3). This label-free approach revealed latent expression structures aligned with lesion severity. Overexpressed miRNAs contributed most to sample separation and were further examined in subsequent analyses. Redundancy was further minimized by integrating principal component loadings with correlation analysis, excluding miRNAs with highly similar expression behavior (Supplementary Fig. S3).

**Fig. 3:**
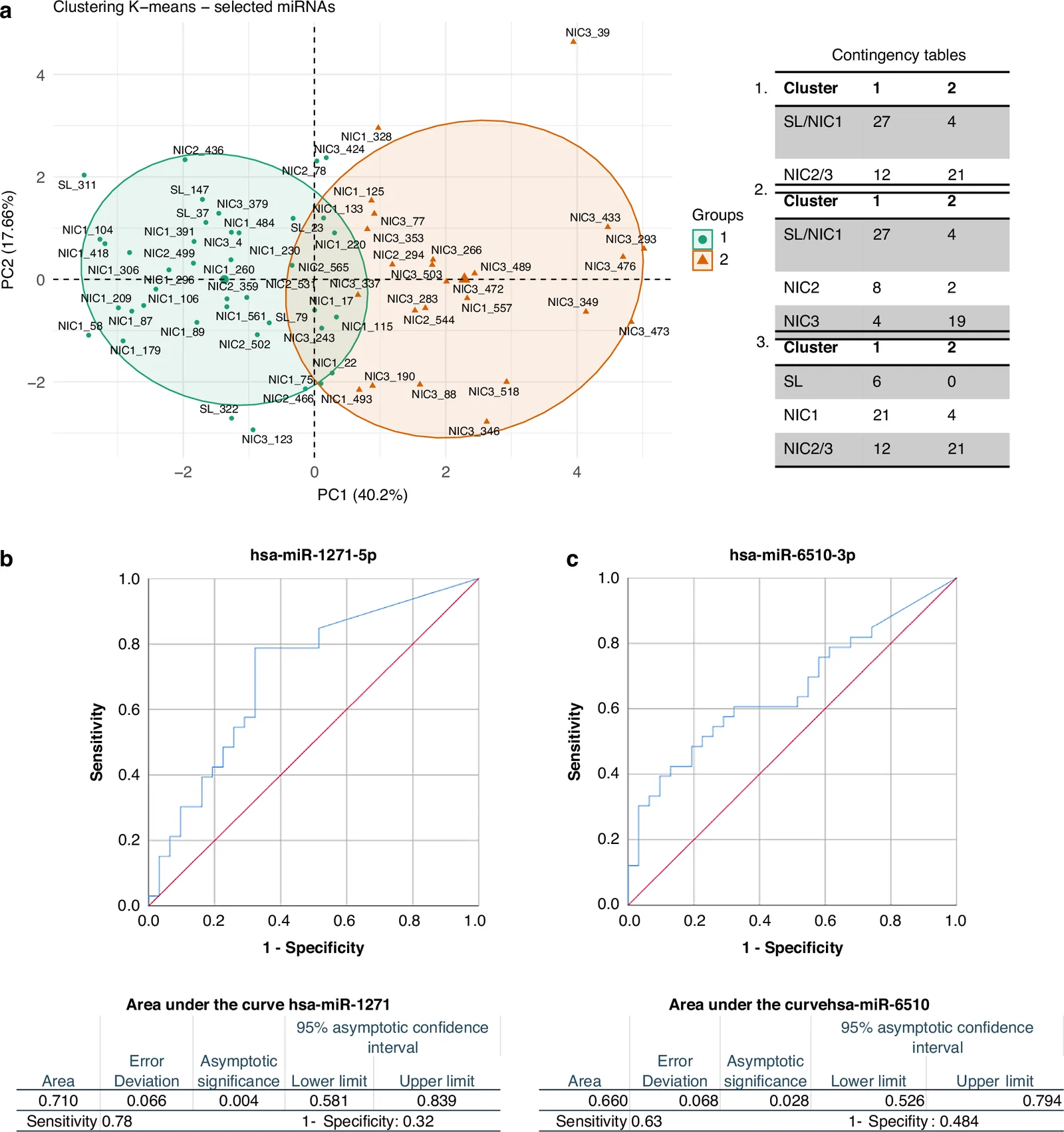
K-means clustering of cervical lesion samples based on miRNA expression. **a** Clustering based on a selected subset of 8 miRNAs improves group discrimination (PC1: 40.2%, PC2: 17.2%). Confidence ellipses and contingency tables illustrate enhanced alignment with clinical diagnosis. **b** hsa-miR-1271 achieved an AUC of 0.710, with a sensitivity of 78% and specificity of 68%, indicating moderate diagnostic performance. Similarly, **c** hsa-miR-6510-3p showed an AUC of 0.660, with sensitivity and specificity values of 63% and 52%, respectively.

Clustering analysis revealed eight miRNAs contributing most prominently to samples stratification: hsa-miR-1271-5p, hsa-miR-9-5p, hsa-miR-501-3p, hsa-miR-1246, hsa-miR-4662a-5p, hsa-miR-3690, hsa-miR-6510-3p, and hsa-miR-184 (Fig. 3a). While clustering was not absolute, a trend emerged: one group was predominantly composed of CIN2/3 samples, and the other of NL and CIN1 cases. As expected, CIN2 samples showed partial overlap due to their intermediate and heterogeneous nature.

To assess individual diagnostic performance, ROC curves analysis was conducted. Only hsa-miR-1271-5p and hsa-miR-6510-3p achieved AUC values above 0.60 with statistical significance (Fig. 3b, c), suggesting moderate discriminatory capability. These findings indicate that the magnitude of differential expression does not uniformly translate into equivalent discriminatory performance. The complete ROC results for all evaluated miRNAs are provided in Supplementary Table S4. Notably, reapplying clustering with just these two miRNAs yielded a separation pattern comparable to that of the full eight-miRNA panel, reinforcing their potential as minimal yet effective markers for distinguishing cervical lesion severity (Supplementary Fig. S4).

We performed a univariate logistic regression analysis to explore potential associations between clinicopathological variables, miRNA expression, and the presence of high-grade cervical lesions (NIC2/3). Among the variables analyzed, two showed statistically significant associations with high-grade lesions: the use of hormonal contraceptives (OR = 5.20, 95% CI: 1.25–21.57, *p* = 0.023) and the expression of hsa-miR-1271-5p (OR = 1.252, 95% CI: 1.171–1.464, *p* = 0.005). Consistent with ROC analysis, miR-1271-5p was the only miRNA demonstrating a statistically significant association with high-grade lesions. Full results are presented in Supplementary Table S5.

Based on the overall results, miRNAs selected for RT-qPCR validation were those showing the most consistent support across the different analytical approaches performed. In particular, hsa-miR-1271-5p, hsa-miR-9-5p, and hsa-miR-501-3p were prioritized, as they were recurrently identified across analyses and also exhibited the highest fold changes in differential expression. This convergence between strong effect size and consistent discriminatory signals supported their selection for validation. Additionally, hsa-miR-4662a-5p was included to represent a candidate with more limited support across analyses, thereby broadening the evaluation spectrum. Finally, hsa-miR-99a-5p was incorporated based on its differential expression, despite limited support from complementary analyses, in order to assess whether differential expression alone could justify experimental validation.

#### Role miRNA in cervical carcinogenesis

**Fig. 4:**
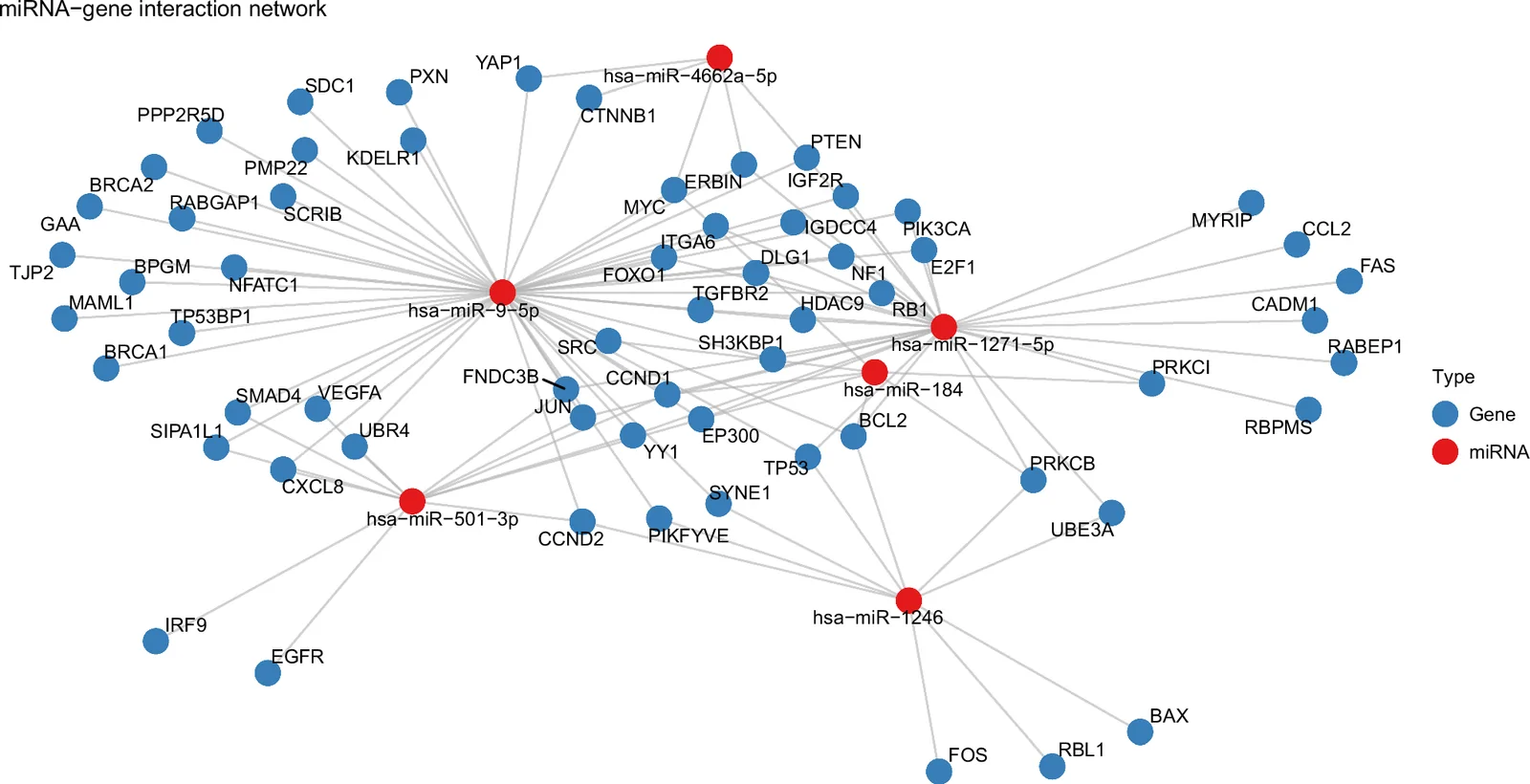
miRNA–target gene interaction network in cervical cancer. The figure shows the interaction network between the eight miRNAs with the greatest discriminatory capacity and the relevant target genes in cervical cancer, identified from a literature review.

The target genes of the miRNAs with the greatest discriminatory capacity between high- and low-grade cervical lesions (hsa-miR-1271-5p, hsa-miR-9-5p, hsa-miR-3690, hsa-miR-6510-3p, hsa- miR-4662a-5p, hsa-miR-184, hsa-miR-501-3p, and hsa-miR-1246) were identified, yielding a total of 7080 target genes. To explore their potential involvement in HPV-induced carcinogenesis, a literature review was conducted focusing on key genes associated with cervical cancer [21–24]. From this search, 71 relevant genes were selected (see Supplementary Table S6), which were evaluated to determine whether they were potentially regulated by the identified miRNAs. The analysis revealed that five of the eight miRNAs regulate key genes such as TP53, CDKN1A, PTEN, MYC, E2F, and FOXO, which are involved in fundamental pathways such as cell cycle regulation, apoptosis, and cell proliferation, which are commonly altered by HPV viral oncoproteins (Fig. 4).

Functional enrichment analysis using gene ontologies (GO) showed a significant representation of biological processes such as “histone modification,” “cell cycle checkpoint,” and “response to DNA damage stimulus.”(Supplementary Fig. S5-A) Regarding molecular functions (Supplementary Fig. S5-B), categories such as “transcription factor binding” and “GTPase activity” stood out, while at the cellular component level (Supplementary Fig. S5-C), structures such as “chromosomal region” and “nucleoplasm” stood out. KEGG pathway analysis indicated that the target genes are involved in critical pathways related to tumor transformation, including the cell cycle, the p53 signaling pathway, proteoglycans in cancer, and human papillomavirus infection, suggesting a functional role for this miRNA network in key processes of cervical cancer progression and development (Supplementary Fig. S5-D).

### Experimental validation

**Fig. 5:**
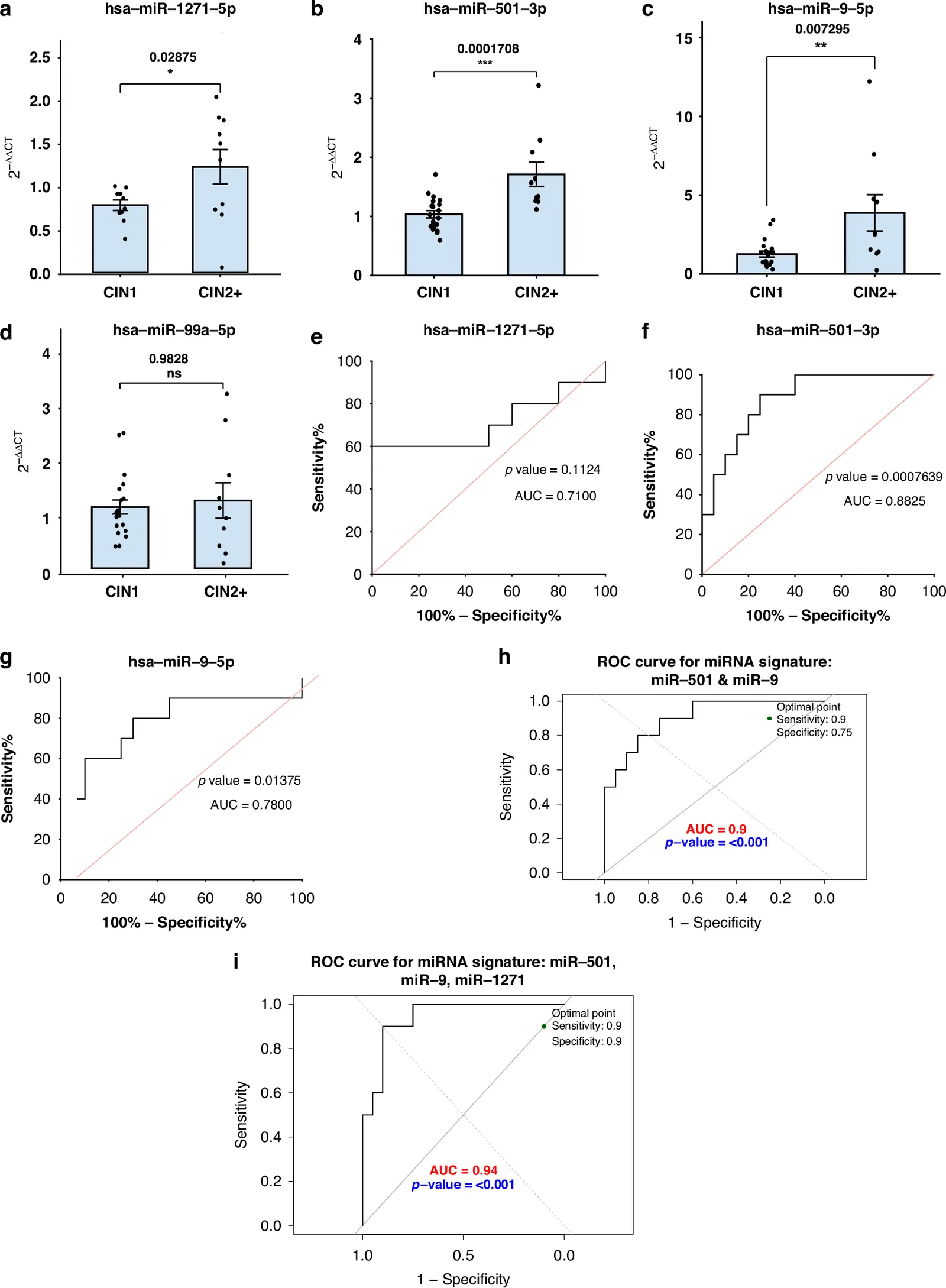
Relative miRNA expression in CIN1 and CIN2+ cervical lesions (CIN2, CIN3). qPCR showed higher expression of (**a**) hsa-miR-1271-5p, **b** hsa-miR-501-3p and **c** hsa-miR-9-5p in CIN2+ (Mann–Whitney U, *p* < 0.05). **d** hsa-miR-99a-5p with no significant differences. ROC curves for (**e**) hsa-miR-1271-5p, **f** hsa-miR-501-3p, and **g** hsa-miR-9-5p were generated from qPCR data. hsa-miR-501-3p showed the best individual performance (AUC = 0.88). **h** Combining hsa-miR-501-3p and hsa-miR-9-5p improved the AUC to 0.90, **i** and adding hsa-miR-1271-5p further increased it to 0.94, with 90% sensitivity and specificity.

To validate the discovery-phase findings, RT-qPCR was performed on an independent cohort of 30 cervical scrape samples from HPV-positive women. hsa-miR-4662a-5p was excluded due to undetermined Ct values in 13 samples. Among the remaining candidates, hsa-miR-1271-5p, hsa-miR- 501-3p, and hsa-miR-9-5p showed significantly higher expression in CIN2+ compared to CIN1 (*p* < 0.05), aligning with small RNA-seq results and supporting their potential as markers for lesión severity (Fig. 5a–c). In contrast, hsa-miR-99a-5p did not show significant differences (Fig. 5d).

Further stratification by lesion grade (CIN1, CIN2, CIN3) revealed significant differences in miRNA expression patterns (Kruskal-Wallis *p* < 0.05). hsa-miR-501-3p and hsa-miR-9-5p displayed greater expression shifts between CIN1 and CIN3, suggesting a role in identifying advanced precancerous changes. Conversely, hsa-miR-1271-5p differed significantly between CIN1 and CIN2 but not between CIN1 and CIN3, potentially indicating involvement in early-stage progression (Supplementary Fig. S6). However, the small number of CIN2 and CIN3 cases (*n* = 5 each) may have limited statistical power.

Diagnostic performance was assessed using ROC curves. hsa-miR-501-3p emerged as the top individual marker (AUC = 0.88; 90% sensitivity; 75% specificity) (Fig. 5e). A combination of hsa-miR-501-3p and hsa-miR-9-5p improved the AUC to 0.90 (Fig. 5h), and inclusion of hsa-miR-1271-5p further increased it to 0.94 with both sensitivity and specificity reaching 90% (Fig. 5i). These results highlight the potential of a multi-miRNA panel for effective triage of HPV-positive women, though validation in larger and more diverse cohorts remains necessary.

## Discussion

HR-HPV screening is highly sensitive for detecting cervical precancerous lesions, but its clinical specificity remains limited due to the high prevalence of transient infections [25]. This has prompted efforts to identify additional molecular markers to improve risk stratification. miRNAs have emerged as potential candidates, given their role in regulating pathways altered by HPV oncogenes E6 and E7, including cell proliferation, apoptosis, and immune evasion [11, 25–27], as well as their high stability, ease of detection in clinical samples, and potential as biomarkers.

Our study provides evidence that cervical scrapes, commonly collected during routine HPV screening, can be successfully used for miRNA profiling through small RNA sequencing. This approach offers a methodological advantage by reflecting real-world sample acquisition, in contrast to biopsy-based studies, and represents, to our knowledge, the first application of this technology in cervical scrapes obtained through routine screening, which are minimally invasive and more easily implementable in clinical programs. Furthermore, we confirmed that cervical scrapes preserved over time remain suitable for miRNA sequencing, consistent with the reported stability of miRNAs across varying RNA integrity values and platforms [28–30], as sequencing performance was comparable despite RIN variability.

The differential expression analysis identified 19 miRNAs between low-grade (≤CIN1) and high- grade (CIN2/3) lesions. Of these, 14 were consistent across internal comparisons. Four miRNAs (hsa-miR-1246, hsa-miR-9-5p, hsa-miR-184, and hsa-miR-99a-5p) displayed concordant expression in independent datasets [12, 13], addressing a critical challenge in the field: the reproducibility of miRNA signatures across studies with varying platforms, extraction methods, and normalization strategies [29], one of the main barriers for the clinical translation of miRNA-based biomarkers.

Despite this, unsupervised clustering based on global miRNA expression revealed limited discrimination between lesion grades, also observed in prior studies [31]. This may reflect heterogeneity in CIN2 lesions, influenced by HPV genotype, viral integration status, and tissue architecture [13, 31–33], and supports proposals to consider a two-tiered classification (low vs. High grade) rather than the traditional three-grade CIN system. To refine discriminatory performance, we restricted the clustering to the most differentially expressed miRNAs, identifying a subset with minimal redundancy and greater stratification potential, prioritizing those overexpressed in CIN2/3.

Notably, most informative miRNAs were overexpressed in CIN2/3 samples, indicating a strong association with high-grade lesions. In contrast, downregulated miRNAs showed lower discriminatory performance in our analyses and were therefore not prioritized within the high-grade biomarker model.

Importantly, reduced discriminatory capacity does not preclude biological relevance, and both upregulated and downregulated miRNAs may participate in regulatory networks involved in cervical carcinogénesis [31]. Their functional roles warrant investigation in future studies specifically designed for that purpose. Among the overexpressed miRNAs, eight were highlighted during the clustering phase as contributing most strongly to sample stratification, and two (hsa-miR-1271-5p and hsa-miR-6510-3p) showed significant AUC values in ROC analysis. Notably, hsa-miR-1271-5p emerged as one of the most consistent candidates from the discovery phase, displaying persistent overexpression in high-grade lesions. Interestingly, miR-1271 has been reported in several cancer types as a tumor suppressor, where it inhibits proliferation and invasion. This expression pattern therefore represents an apparently contradictory finding. We hypothesize that this pattern may reflect the context-dependent nature of miRNA regulation, which can vary according to tissue type, stage of disease, HPV-associated molecular alterations, and early transformation dynamics. Given the limited available evidence regarding miR-1271 in cervical lesions, its role in this setting remains to be clarified. Functional studies in cervical-specific models will be required to test these hypotheses.

Functional analyses revealed that several of these miRNAs, including hsa-miR-1271-5p and hsa-miR- 9-5p, target tumor suppressors such as TP53, RB1, and BRCA1 [22]. Additional targets like FOXO1, E2F, and BCL2L11 reinforce their relevance in apoptosis and cell cycle control [22, 23], along with genes identified in this study such as BRCA2, PIK3CA, RBL1, FOS, BAX, MYCN, KRAS, VEGF, and TP53INP1. Moreover, previous research has shown that HPV-derived miRNAs can modulate host genes involved in cell cycle, migration, and immune response. Enrichment analysis supported this by highlighting pathways such as p53 signaling, DNA repair, and PI3K-Akt signaling [34]. However, it is worth noting the complex regulatory architecture of miRNA networks, where single miRNAs can target multiple genes, and vice versa. While functional validation remains necessary, the utility of miRNAs as biomarkers lies primarily in their reproducible association with clinical states [35].

Logistic regression supported the association between high-grade lesions and two miRNAs: hsa-miR-1271-5p and hsa-miR-4662a-5p, as well as the use of hormonal contraceptives, echoing prior findings that integrated models combining molecular and epidemiological data may enhance risk prediction [36].

qPCR validation reinforced the overexpression of hsa-miR-501-3p, hsa-miR-9-5p, and hsa-miR-1271-5p in high-grade lesions. Notably, hsa-miR-501-3p achieved an AUC of 0.88, outperforming previously reported panels [37]. Although hsa-miR-501 family members have been implicated in cancer biology, most functional evidence in cervical cancer refers specifically to hsa-miR-501-5p. In cervical carcinoma, hsa-miR-501-5p is consistently upregulated and promotes cell proliferation, migration, and invasion through suppression of CYLD and activation of NF-κB pathways [37]. In contrast, hsa-miR-501- 3p has predominantly been associated with tumor-suppressive roles in several cancer types [38]. Some studies report tumor-suppressive action, such as downregulation and inhibition of tumor growth in non- small cell lung cancer, where overexpression of miR-501-3p suppresses cell proliferation and invasión [39]. Other studies in osteosarcoma and colorectal cancer demonstrate upregulation of miR-501-3p and promotion of invasive phenotypes [40], indicating that this miRNA can exhibit oncogenic features depending on cellular context.

At present, there are no functional studies specifically characterizing miR-501-3p in cervical neoplasia. Therefore, while our findings support its diagnostic utility in CIN2+ lesions, further experimental validation is pending. hsa-miR-9-5p also demonstrated high performance and has been consistently associated with cervical cancer progression in previous studies, acting on target FOXO3, reinforcing its link to cervical progression [41, 42]. In contrast, hsa-miR-1271-5p did not reach statistical significance in ROC analysis, showed a trend toward increased expression in CIN2, suggesting a role in early transformation [43]. hsa-miR-1271 has been reported predominantly as a tumor suppressor in multiple cancer types, where it is often downregulated [43]. The molecular mechanisms of miR-1271 suggest its involvement in tumor suppression and regulation of oncogenic pathways across multiple tissues, indicating a context-dependent role in neoplastic progression [43]. For instance, differential upregulation has been described in certain non-malignant proliferative conditions, indicating that its expression pattern may vary according to the cellular microenvironment [44]. The expression patterns observed for miR-501-3p and miR-1271-5p highlight the complexity of miRNA regulation in preneoplastic cervical lesions. Although often described as tumor suppressors in invasive malignancies, their increased expression in high-grade lesions suggests stage-specific and context-dependent roles. As this study is limited to expression profiling, functional implications cannot yet be established; however, these findings support the concept that miRNA dysregulation is a dynamic process during cervical carcinogenesis. Longitudinal analyses including invasive carcinoma samples will be necessary to clarify whether these alterations represent early adaptive responses or progression-associated shifts.

The combination of these three miRNAs achieved an AUC of 0.94 with 90% sensitivity and specificity, indicating that multivariate models may outperform individual markers, as reported in previous triage studies [11–13]. Although small RNA-seq offers a broad overview of the transcriptome, isomiR variants could cause discrepancies with qPCR; this did not appear to affect our results, but future studies should explore this aspect. It is also worth mentioning that qPCR detected all target miRNAs efficiently, with stable expression of endogenous control [45].

In summary, the identified miRNAs, especially when combined, offer promising tools for improving triage strategies and risk stratification among women positive for high-risk HPV. The high diagnostic performance of this miRNA panel (AUC = 0.94, 90% sensitivity and specificity) indicates that it can reliably discriminate between low- and high-grade lesions, supporting its potential use as a secondary test following a positive HR-HPV result. Their detection in cervical scrapes, routinely collected during HPV screening, demonstrates that this type of sample is suitable for biomarker identification and allows direct integration into existing clinical workflows without additional sampling., A triage algorithm based on these miRNAs, assessed by qPCR, would enable patient classification according to risk: those with expression profiles indicative of high-grade lesions would be prioritized for immediate colposcopy, while low-risk patients would remain under regular surveillance, optimizing risk stratification and clinical resource allocation. This approach would reduce unnecessary procedures and focus clinical attention on patients most likely to harbor or develop high-grade cervical lesions, particularly in settings with limited access to specialized diagnostic services. Further validation in prospective multicenter cohorts will be required before widespread clinical adoption.

### Limitations

While our study provides important insights into the potential of miRNAs for risk stratification in women positive for high-risk HPV, certain aspects warrant consideration. First, the sample size, particularly in the validation cohort, was relatively limited; expanding patient cohorts in future studies would allow more precise evaluation of diagnostic performance and clinical utility. Additionally, cervical intraepithelial neoplasia grade 2 (CIN2) lesions are biologically heterogeneous, which may influence miRNA expression patterns; further comparative analyses could help clarify potential variability within this diagnostic category. Although our findings were internally validated, multicenter prospective studies in larger and more diverse populations will be necessary to confirm reproducibility and generalizability. Furthermore, while cervical scrapes stored over several years proved suitable for miRNA profiling, prospective analyses using newly collected samples may further optimize robustness. From a translational perspective, future research may explore whether miRNA expression patterns differ according to specific high-risk HPV genotypes, potentially refining biomarker specificity. Moreover, miRNAs identified during the Discovery phase but not validated, as well as those found to be downregulated, deserve further investigation in independent cohorts and functional assays to better elucidate their role in cervical carcinogenesis and their potential clinical relevance.

## Supplementary information

Supplementary_Materials_Figures_and_Tables.

Supplementary file 1. Enrichment_with_target miRNAs

## Data Availability

The small RNA sequencing data have been deposited in the Gene Expression Omnibus (GEO) under accession number GSE325355 and will be made publicly available upon publication. The code used for differential expresión análisis is available at: https://github.com/lmejiagu-lang/miRNA_cervical_cancer_analysis.
