# Supplementary material for: Identification of microRNAs as biomarkers for triage of cervical intraepithelial neoplasia in cervical scrape samples from high-risk HPV-positive women
Source: Br J Cancer. 2026 Aug 4;135(9):1524–34. doi: 10.1038/s41416-026-03521-y (PMC13675211; doi:10.1038/s41416-026-03521-y)
Supplement: Supplementary file 1 [file 41416_2026_3521_MOESM1_ESM.pdf]

SUPPLEMENTARY FIGURE LEGENDS

Supplementary Figure S1. Exploratory analysis of miRNA expression across cervical lesión stages. Venn diagram showing the differentially expressed miRNAs identified in the comparisons between NL (no lesion) vs. CIN1, NL vs. CIN2/3, and CIN1 vs. CIN2/3. Exclusive miRNAs for each comparison are indicated Upregulated miRNAs are shown in red and downregulated in blue, while 13 shared miRNAs (hsa-miR-1271-5p, miR-9-5p, miR-501-3p, miR-221-5p, miR-99a-5p, miR-1246, miR-4662a-5p, miR-96-5p, miR-422a, miR-193b-5p, miR-29b-2-5p, miR-365a-5p, hsa-miR-320b and miR-210-3p) suggest stage-specific expression patterns and potential involvement in lesión progression. The table shows the differentially expressed miRNAs along with their significance values and Foldchange.

Supplementary Figure S2. Overlap of differentially expressed miRNAs identified in this study with those reported in two independent public datasets (GSE104758 and GSE167858). Shared miRNAs are highlighted, supporting consistency across cohorts. The table shows summary of Re-Analyzed Datasets GEO (Gene Expression Omnibus) NCBI.

Supplementary File 1. Enrichment with target miRNAs differential expression

Supplementary Figure S3. (A) Principal component analysis (PCA) based on normalized expresión values of the selected miRNAs. The upper plot shows the contribution of each miRNA to the first two principal components (Dim1: 27.5% and Dim2: 21.7%). Arrows indicate the direction and weight of each variable in the separation of samples, with the color gradient representing their level of contribution. The lower plot displays the distribution of samples in the PCA space, highlighting which miRNAs may be driving group separation. (B) Correlation matrix of the miRNAs included in the analysis. Pearson correlation coefficients between expression levels are shown, with circle size and color intensity indicating the strength and direction of the correlations. Distinct clusters of highly correlated miRNAs were identified, suggesting potential functional relationships or co-expression. This information complemented the PCA analysis and contributed to the prioritization of candidate miRNAs by highlighting those with robust expression patterns, informative redundancy, and relevance in group discrimination.

Supplementary Figure S4. K-means clustering of cervical lesion samples based on the expresión of hsa-miR-1271 and hsa-miR-6510. Samples were grouped into two clusters according to their microRNA expression profiles using the K-means algorithm. Cluster 2 shows a clear tendency to group high-grade lesions (NIC2/3), with only a small number of low-grade lesions (SL/NIC1) included in this cluster.

Supplementary Figure S5. Functional enrichment analysis of target genes regulated by discriminative miRNAs. Bar graphs summarizing significantly enriched terms according to gene ontologies (GO) and KEGG pathways.

Supplementary Figure S6. Relative miRNA expression according to histological grade. Expression of hsa-miR-1271-5p, hsa-miR-501-3p, and hsa-miR-9-5p was assessed by qPCR in CIN1, CIN2, and CIN3 samples. Significant differences (Kruskal-Wallis, $p < 0.05$; Dunn post hoc) were observed: hsa-miR-501-3p and hsa-miR-9-5p between CIN1 and CIN3; hsa-miR-1271-5p between CIN1 and CIN2.

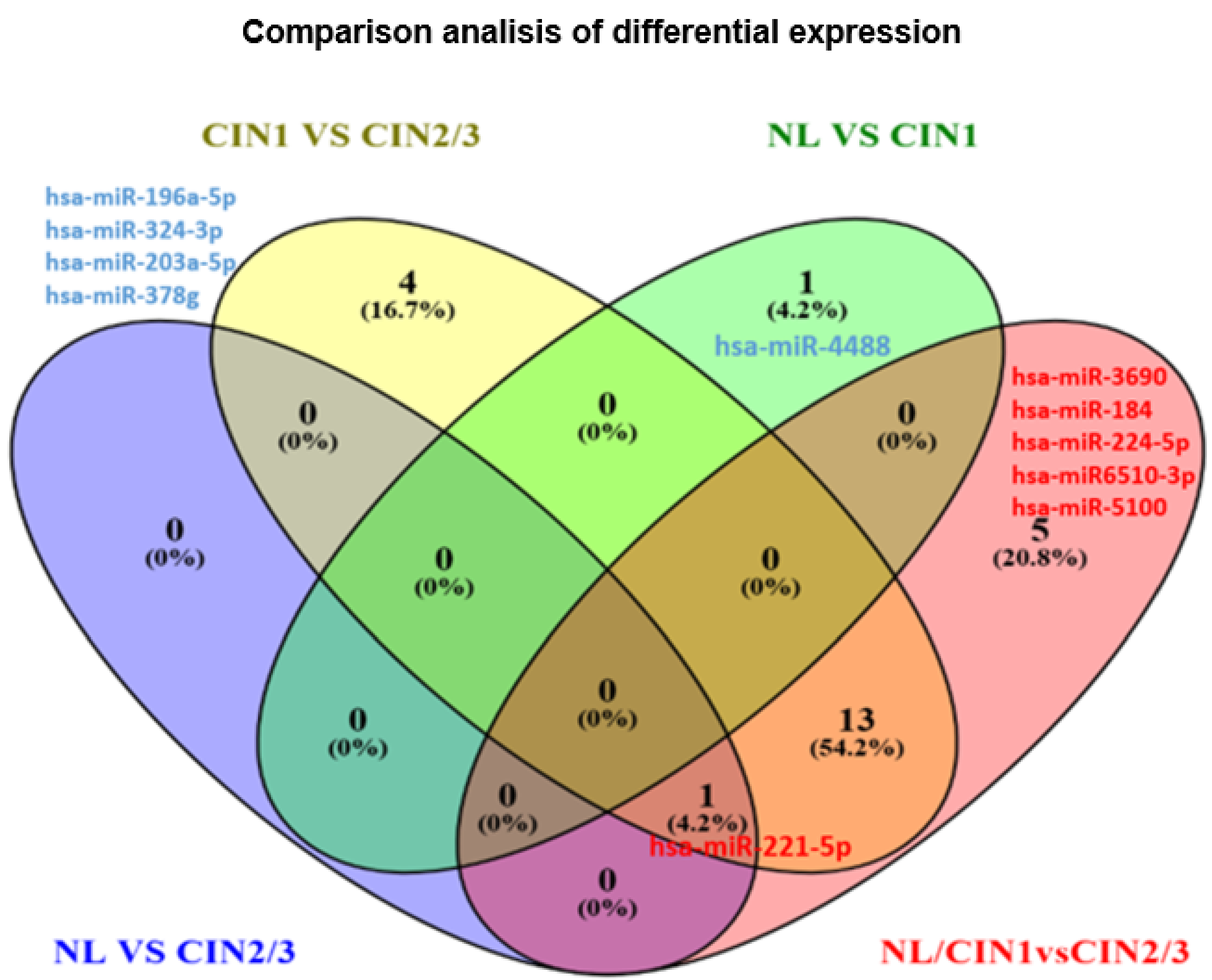

CIN1 vs CIN2/3

	baseMean	FC	log2FC	pvalue	padj
hsa-miR-1271-5p	8.94982188	7.52120024	2.91096291	1.2949E-06	0.00064497
hsa-miR-422a	5.31683657	0.12674716	-2.97997468	2.3582E-06	0.00064497
hsa-miR-1246	11.0663188	5.11810608	2.35561005	4.0109E-06	0.00073133
hsa-miR-9-5p	60.3424202	3.67601169	1.87814136	2.1475E-05	0.00293669
hsa-miR-193b-5p	13.875878	0.26950826	-1.89159858	6.1587E-05	0.00561466
hsa-miR-210-3p	1189.88866	0.31502165	-1.66647713	5.9932E-05	0.00561466
hsa-miR-365a-5p	4.60219046	0.22418416	-2.15724375	0.00014718	0.00925155
hsa-miR-501-3p	167.427746	3.26046045	1.70507572	0.00015222	0.00925155
hsa-miR-324-3p	23.5359336	0.45280466	-1.1430393	0.00038228	0.02091066
hsa-miR-221-5p	50.6261031	2.11251849	1.07896397	0.00049182	0.02241874
hsa-miR-99a-5p	491.605881	2.80052528	1.48569745	0.00048036	0.02241874
hsa-miR-29b-2-5p	3.5562059	0.47154602	-1.0845295	0.00058539	0.02310704
hsa-miR-96-5p	38.6010099	2.52176123	1.33443168	0.00063365	0.02310704
hsa-miR-4662a-5p	3.67407338	2.6460463	1.40383831	0.00072309	0.02472065
hsa-miR-196a-5p	25.3008194	2.05731702	1.04076412	0.00092964	0.02825077
hsa-miR-203a-5p	14.0709267	0.23060042	-2.11653293	0.00090254	0.02825077
hsa-miR-320b	430.58174	0.47913522	-1.06149521	0.00141724	0.04080159
hsa-miR-378g	4.08076636	0.49377745	-1.01806714	0.00181316	0.04958995

NL vs CIN1

	baseMean	FC	log2FC	pvalue	padj
hsa-miR-4488	4.49156093	0.01657017	-5.91526799	8.478E-07	0.00060533

NL vs CIN2/3

	baseMean	FC	log2FC	pvalue	padj
hsa-miR-221-5p	50.6261031	4.6322253	2.21170542	5.7276E-05	0.04089522

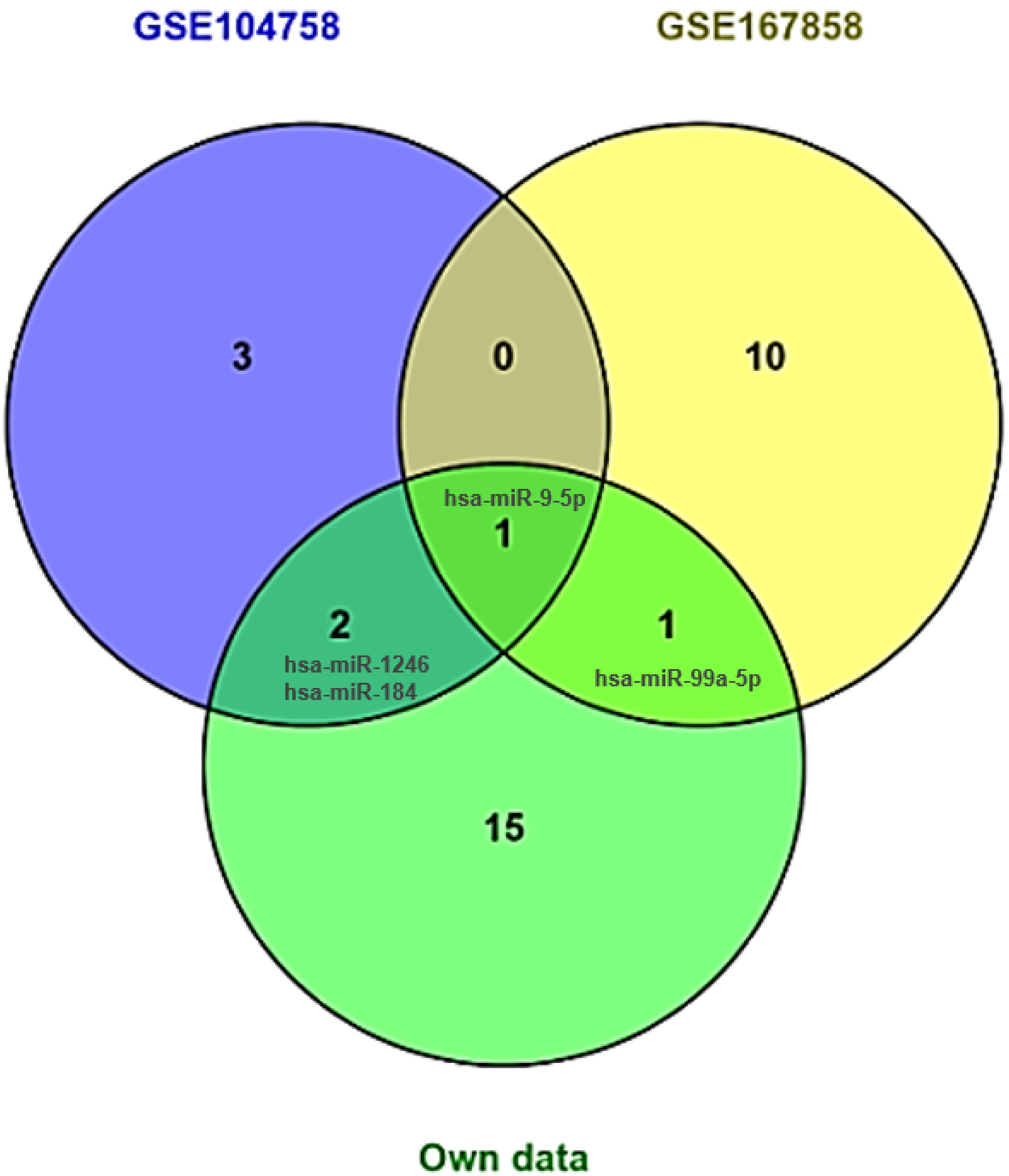

Study access number	Year of study	Title	Platform	Origin of the sample	miRNAs
GSE104758	2018	Genome-wide microRNA analysis of HPV-positive self-samples yields novel triage markers for early detection of cervical cancer.	HiSeq 2500 (Illumina)	Tests for autotoma cervicovaginales positivas para HR-HPV	**UP**:<br>hsa-miR-1246<br>hsa-miR-20a-5p<br>hsa-miR-9-5p<br>hsa-miR-93-5p<br>hsa-miR-184<br>**Down:**<br>hsa-miR-22-3p
GSE167858	2023	miRNAs signature as potential biomarkers for cervical precancerous lesions in Human Papillomavirus Positive women	Illumina MiSeq (Homo sapiens)	Parafina test with preneoplastic cervical lesion and without HR-HPV positive female lesion	hsa-miR-1-3p<br>hsa-miR-133a-3p<br>hsa-miR-133b<br>hsa-miR-143-3p<br>hsa-miR-143-5p<br>hsa-miR-145-3p<br>hsa-miR-145-5p<br>hsa-miR-7976<br>hsa-miR-204-5p<br>hsa-miR-30b-5p<br>hsa-miR-99a-5p<br>hsa-miR-9-5p<br>**Down:** none

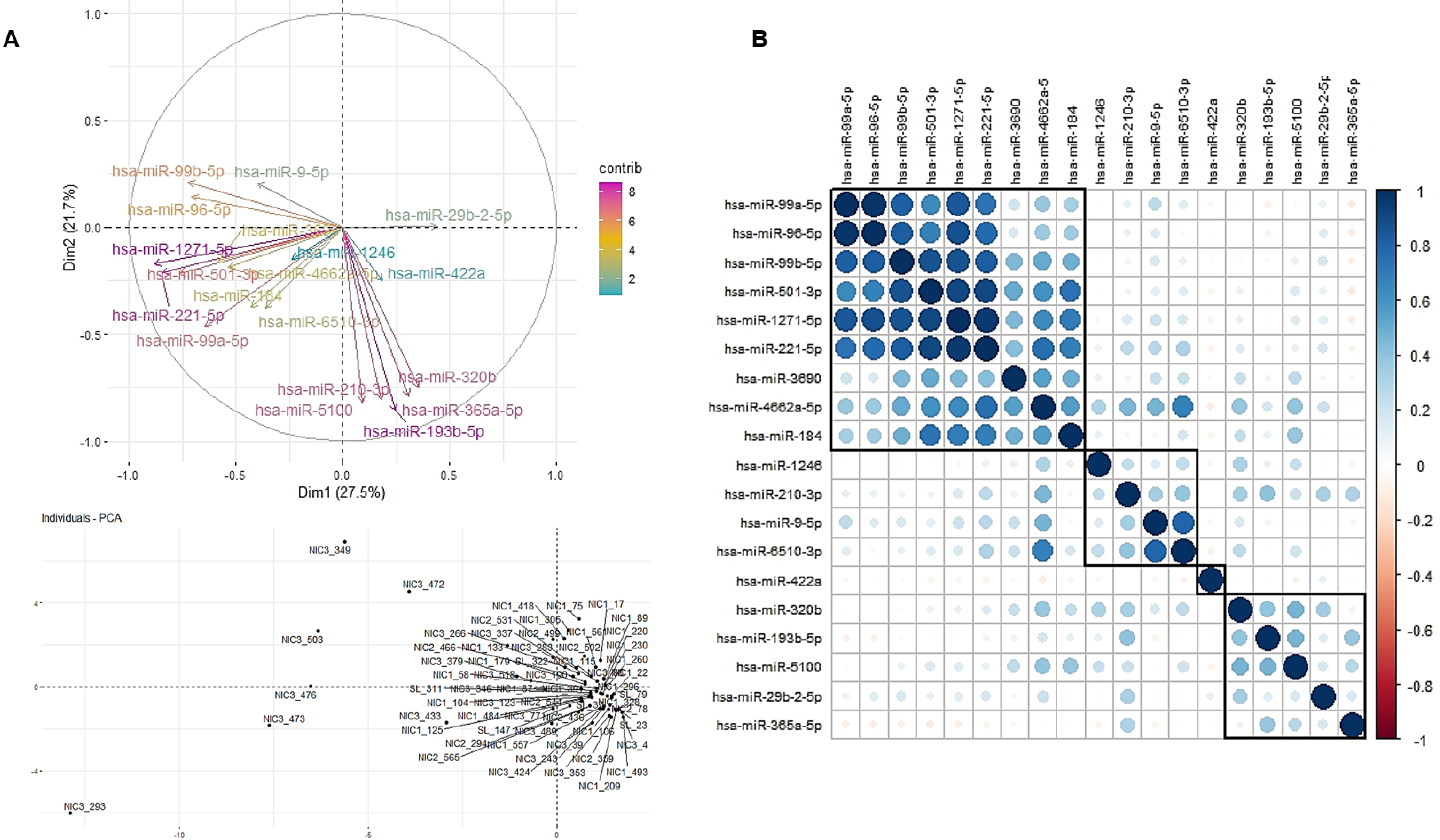
A
B
contrib
8
6
4
2
Dim1 (27.5%)
Dim2 (21.7%)
hsa-miR-99b-5p
hsa-miR-9-5p
hsa-miR-96-5p
hsa-miR-29b-2-5p
hsa-miR-1271-5p
hsa-miR-1246
hsa-miR-501-3p
hsa-miR-4662a-5p
hsa-miR-422a
hsa-miR-184
hsa-miR-221-5p
hsa-miR-6510-3p
hsa-miR-99a-5p
hsa-miR-320b
hsa-miR-210-3p
hsa-miR-5100
hsa-miR-365a-5p
hsa-miR-193b-5p
Individuals - PCA
NIC3_349
NIC3_472
NIC3_503
NIC3_476
NIC3_473
NIC3_293
NIC1_418
NIC1_75
NIC1_17
NIC1_89
NIC3_433
NIC1_125
NIC2_294
NIC2_565
NIC3_424
NIC3_353
NIC1_209
NIC1_493
hsa-miR-99a-5p
hsa-miR-96-5p
hsa-miR-99b-5p
hsa-miR-501-3p
hsa-miR-1271-5p
hsa-miR-221-5p
hsa-miR-3690
hsa-miR-4662a-5p
hsa-miR-184
hsa-miR-1246
hsa-miR-210-3p
hsa-miR-9-5p
hsa-miR-6510-3p
hsa-miR-422a
hsa-miR-320b
hsa-miR-193b-5p
hsa-miR-5100
hsa-miR-29b-2-5p
hsa-miR-365a-5p
1
0.8
0.6
0.4
0.2
0
-0.2
-0.4
-0.6
-0.8
-1

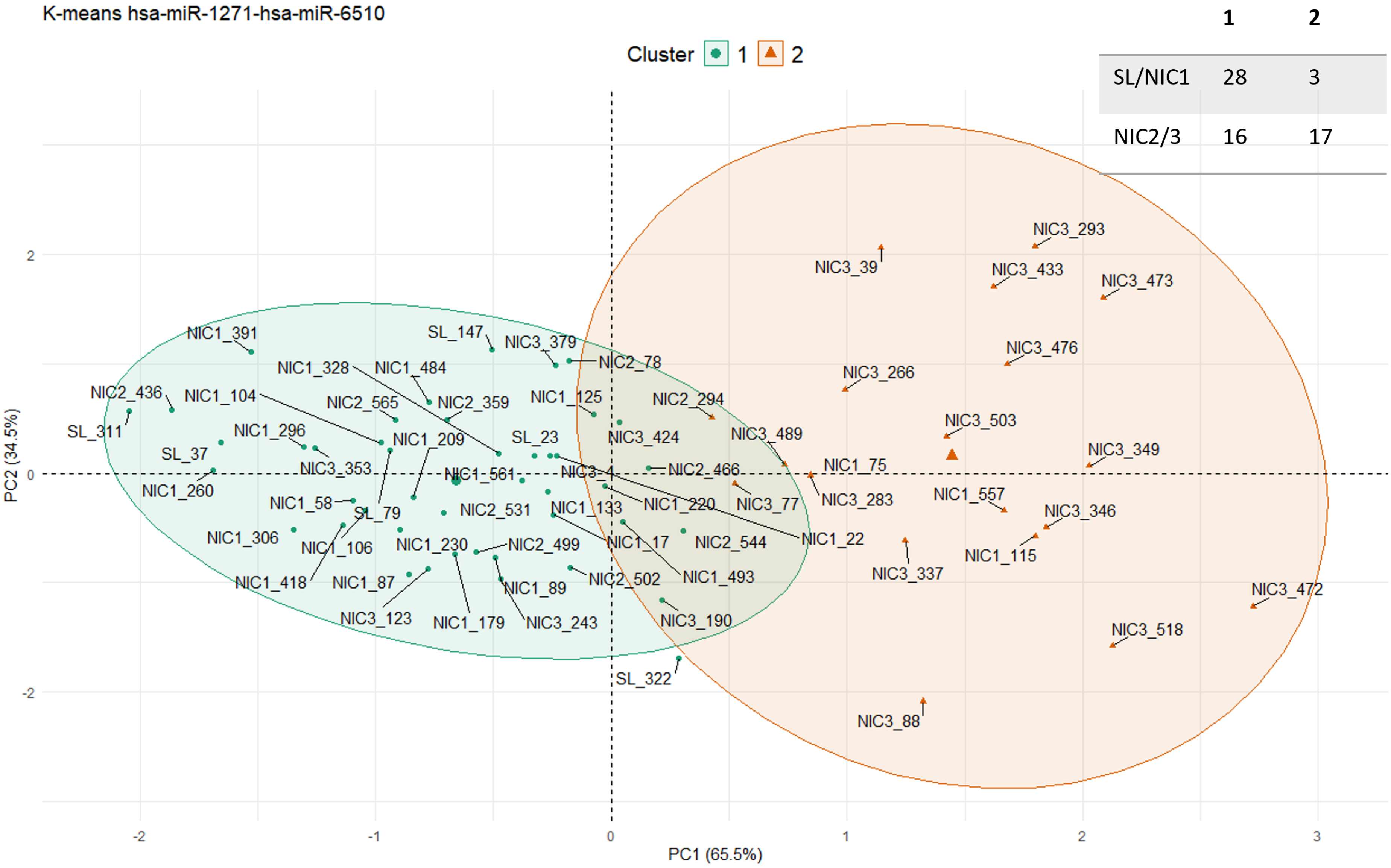

	1	2
SL/NIC1	28	3
NIC2/3	16	17

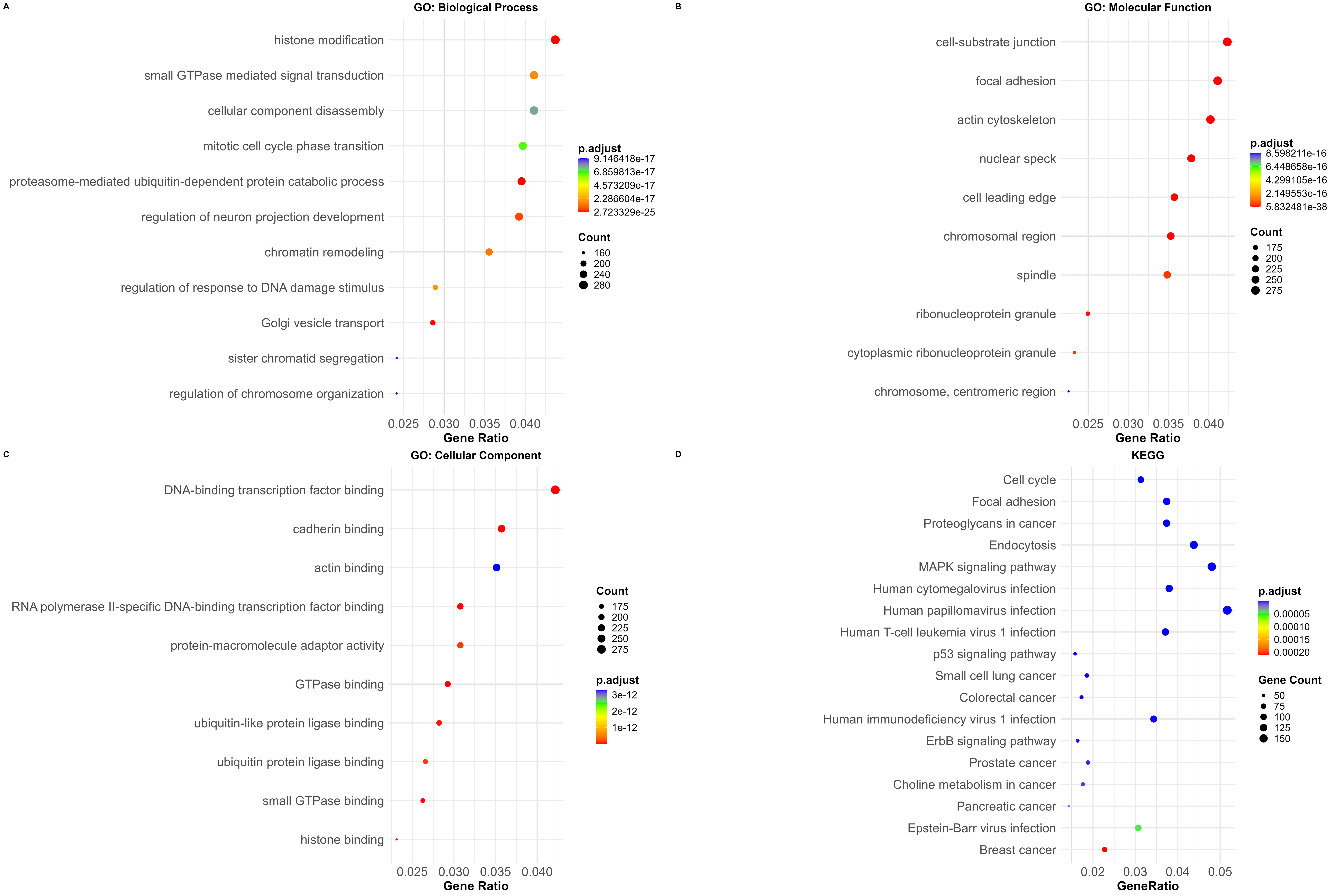
A
GO: Biological Process
histone modification
small GTPase mediated signal transduction
cellular component disassembly
mitotic cell cycle phase transition
proteasome-mediated ubiquitin-dependent protein catabolic process
regulation of neuron projection development
chromatin remodeling
regulation of response to DNA damage stimulus
Golgi vesicle transport
sister chromatid segregation
regulation of chromosome organization
0.025 0.030 0.035 0.040
Gene Ratio
p.adjust
9.146418e-17
6.859813e-17
4.573209e-17
2.286604e-17
2.723329e-25
Count
160
200
240
280
B
GO: Molecular Function
cell-substrate junction
focal adhesion
actin cytoskeleton
nuclear speck
cell leading edge
chromosomal region
spindle
ribonucleoprotein granule
cytoplasmic ribonucleoprotein granule
chromosome, centromeric region
0.025 0.030 0.035 0.040
Gene Ratio
p.adjust
8.598211e-16
6.448658e-16
4.299105e-16
2.149553e-16
5.832481e-38
Count
175
200
225
250
275
C
GO: Cellular Component
DNA-binding transcription factor binding
cadherin binding
actin binding
RNA polymerase II-specific DNA-binding transcription factor binding
protein-macromolecule adaptor activity
GTPase binding
ubiquitin-like protein ligase binding
ubiquitin protein ligase binding
small GTPase binding
histone binding
0.025 0.030 0.035 0.040
Gene Ratio
Count
175
200
225
250
275
p.adjust
3e-12
2e-12
1e-12
D
KEGG
Cell cycle
Focal adhesion
Proteoglycans in cancer
Endocytosis
MAPK signaling pathway
Human cytomegalovirus infection
Human papillomavirus infection
Human T-cell leukemia virus 1 infection
p53 signaling pathway
Small cell lung cancer
Colorectal cancer
Human immunodeficiency virus 1 infection
ErbB signaling pathway
Prostate cancer
Choline metabolism in cancer
Pancreatic cancer
Epstein-Barr virus infection
Breast cancer
0.02 0.03 0.04 0.05
GeneRatio
p.adjust
0.00005
0.00010
0.00015
0.00020
Gene Count
50
75
100
125
150

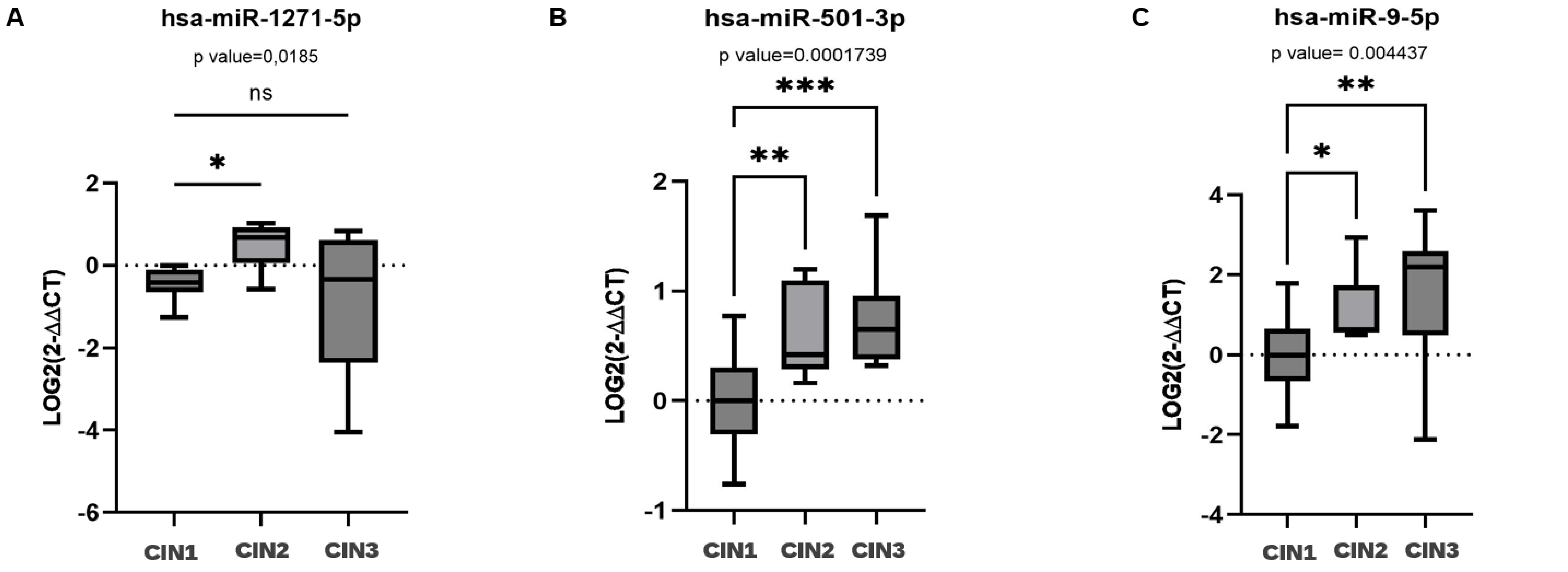
A
hsa-miR-1271-5p
p value=0,0185
ns
*
LOG2(2-ΔΔCT)
2
0
-2
-4
-6
CIN1
CIN2
CIN3
B
hsa-miR-501-3p
p value=0.0001739
***
**
LOG2(2-ΔΔCT)
2
1
0
-1
CIN1
CIN2
CIN3
C
hsa-miR-9-5p
p value= 0.004437
**
*
LOG2(2-ΔΔCT)
4
2
0
-2
-4
CIN1
CIN2
CIN3

Supplementary Table S1. Clinicopathological and Sociodemographic Characteristics of the Study Population by Lesion Grade (NL/CIN1 vs. CIN2/3) discovery group.

Sociodemographic Variable	SL/NIC1		NIC2/3		All		p-value
	n	%	n	%	n	%	
**Age (years)**							
Under 30	16	61.54	10	38.46	26	36.11	0.13
Over 30	15	39.47	23	60.53	38	52.78	
**Occupation**							
Housewife	7	36.84	12	63.16	19	26.39	
Technician	1	33.33	2	66.67	3	4.17	0.52
Professional	9	50	9	50	18	25	
Other	14	58.33	10	41.67	24	33.33	
**Education**							
Primary	3	33.33	6	66.67	9	12.5	
Secondary	7	50	7	50	14	19.44	0.66
Technical	5	41.67	7	58.33	12	16.67	
University	16	55.17	13	44.83	29	40.28	
**Socioeconomic Level**							
Low	3	33.33	6	66.67	9	12.5	
Medium	25	48.08	27	51.92	52	72.22	0.13
High	3	100	0	0	3	4.17	
**Number of Births**							
0	11	57.89	8	42.11	19	26.39	
1-2	13	54.17	11	45.83	24	33.33	0.23
3+	7	33.33	14	66.67	21	29.17	
**Sexual Partners**							
1-2	9	40.91	13	59.09	22	30.56	0.54
3+	22	52.38	20	47.62	42	58.33	
**Contraceptive Use**							
None	12	70.59	5	29.41	17	23.61	
DIU	3	33.33	6	66.67	9	12.5	
Hormonal	9	45	11	55	20	27.78	0.29
Tubal Ligation (Pomeroy)	4	40	6	60	10	13.89	
Condom	3	37.5	5	62.5	8	11.11	
**Duration of Contraceptive Use**							
1-48 months	26	63.41	15	36.59	41	56.94	0.16
60 to 230 months	5	21.74	18	78.26	23	31.94	
**Smoking**							
Never	24	50	24	50	48	66.67	
Smoker	2	33.33	4	66.67	6	8.33	0.73
Quit	5	50	5	50	10	13.89	

*The chi-square test was applied.

Supplementary Table S2. Clinicopathological and Sociodemographic Characteristics of the Study Population by Lesion Grade (NL/CIN1 vs. CIN2/3) validation group.

Variable	NIC1		NIC2/3		Todos		Valor p*
	n	%	n	%	n	%	
**Age (years)**							
Under 30	10	83.33	2	16.67	12	16.67	0.23
Over 30	10	55.56	8	44.44	18	25	
**Occupation**							
Housewife	6	75	2	25	8	11.11	0.10
Technician	0	0	3	100	3	4.17	
Professional	4	80	1	20	5	6.94	
others	10	71.43	4	28.57	14	19.44	
**Education**							
Primary	1	50	1	50	2	2.78	0.22
secondary	9	75	3	25	12	16.67	
Technical	2	33.33	4	66.67	6	8.33	
University	8	80	2	20	10	13.89	
**Socioeconomic Level**							
Low	7	58.33	5	41.67	12	16.67	0.86
Medium	10	71.43	4	28.57	14	19.44	
High	3	75	1	25	4	5.56	
**Number of Births**							
0	8	80	2	20	10	13.89	0.49
1-2	6	54.55	5	45.45	11	15.28	
3+	6	66.67	3	33.33	9	12.5	
**Sexual Partners**							
1-2	10	76.92	3	23.08	13	18.06	0.51
3+	10	58.82	7	41.18	17	23.61	
**Contraceptive Use**							
None	7	77.78	2	22.22	9	12.5	0.52
DIU	2	50	2	50	4	5.56	
hormonal	3	42.86	4	57.14	7	9.72	
Tubal Ligation (Pomeroy)	3	75	1	25	4	5.56	
Condom	5	83.33	1	16.67	6	8.33	
**Smoking**							
None	18	78.26	5	21.74	23	31.94	0.22
Smoker	1	50	1	50	2	2.78	
Quit	1	20	4	80	5	6.94	

*The Fisher test was applied.

Supplementary Table S3. RNA integrity assessment of samples included in the sequencing analysis. RNA integrity was evaluated prior to library preparation using the Agilent 2100 Bioanalyzer. RNA Integrity Number (RIN) values are reported for each sample as an indicator of RNA quality.

Sample	RIN	Sample	RIN	Sample	RIN	Sample	RIN
22	6.7	230	NA	293	2.6	39	6.8
58	6.8	493	5.2	349	5.5	77	6.3
113	6.1	544	5.3	476	7.5	489	6.3
179	5.1	391	7.9	477	2.1	4	6.2
209	7.7	418	7.5	431	5.2	88	3
104	5.3	466	7.3	433	6.4	123	5.2
565	5	557	NA	473	7.8	190	4.3
75	5.3	484	3.2	503	7.3	518	7.2
89	5	220	5.6	266	NA	79	3.1
260	6.3	564	6.3	277	2.6	147	6.3
502	5	78	NA	379	6	346	2.1
311	5.1	499	6.7	472	6.2	411	7.4
87	6.1	106	6.3	283	5.5	23	2.9
37	6.1	133	5.2	337	3.3	17	NA
561	8.2	296	5.2	353	6.2	531	5.2
322	6.4	306	6.7	424	5.3	125	NA
359	7.2	243	3.2	436	NA	115	3
328	2.4	380	3.6				

Supplementary Table S4. Univariate ROC analysis showing the diagnostic performance of individual miRNAs for distinguishing ≤CIN1 from CIN2/3 lesions. AUC values, standard errors, p-values, and 95% confidence intervals are presented. Only hsa-miR-1271-5p and hsa-miR-6510-3p showed statistically significant discriminatory capacity.

Test result variables	Area	Deviation Error[a]	Asymptotic significance[b]	95% asymptotic confidence interval	
				Lower limit	Upper limit
hsa-miR-1246	0.612	0.071	0.124	0.473	0.751
hsa-miR-1271-5p	0.710	0.066	0.004	0.581	0.839
hsa-miR-184	0.629	0.072	0.077	0.488	0.769
hsa-miR-221-5p	0.454	0.073	0.528	0.311	0.597
hsa-miR-3690	0.624	0.070	0.088	0.486	0.762
hsa-miR-4662a-5p	0.615	0.070	0.113	0.477	0.753
hsa-miR-501-3p	0.445	0.073	0.448	0.301	0.588
hsa-miR-6510-3p	0.660	0.068	0.028	0.526	0.794
hsa-miR-9-5p	0.615	0.071	0.114	0.476	0.754
hsa-miR-96-5p	0.507	0.074	0.925	0.362	0.651
hsa-miR-99a-5p	0.579	0.072	0.280	0.438	0.719
hsa-miR-99b-5p	0.397	0.072	0.156	0.256	0.538

a. Under the non-parametric assumption
b. Null hypothesis: true area = 0.5

Supplementary Table S5. Univariate logistic regression analysis for High grade cervical lesions.

**Clinical characteristics**	**P value**	**OR**	**95% CI lower**	**95% CI higher**
**Occupation**				
Homemaker (Ref)				
Technician or Professional	0.433	0.615	0.183	2.072
Other	0.661	0.769	0.238	2.488
**Education**				
Primary and secondary (Ref)				
Technical and/or University	0.942	0.963	0.346	2.674
**Socioeconomic Level**				
1+2 (Ref) (Low)				
3 (Medium)	0.565	0.64	0.14	2.921
4+5+6 (High)	0.772	0.8	0.177	3.618
**Number of Births**				
1 o 2 (Ref)	0.358	1.806	0.512	6.363
+ 2	0.283	1.92	0.583	6.324
**Sexual Partners**				
Hasta 2 (Ref)				
+ 2	0.384	0.629	0.222	1.787
**Contraceptive Use**				
None (Ref)				
Hormonal	**0.023**	5.2	1.253	21.572
Others	0.311	1.878	0.555	6.358
**Duration of Contraceptive Use**				
Does not use (Ref)				
From 1 to 48 months	**0.029**	4.5	1.166	17.373
From 60 to 230 meses	0.441	1.587	0.49	5.138
**Smoking**				
None (Ref)				
Smoke or have smoked in the past	0.472	1.519	0.486	4.748
**hsa-miR-1246**	0.056	1.051	0.999	1.106
**hsa-miR-1271-5p**	0.005	1.252	1.171	1.464
**hsa-miR-3690**	0.024	1.053	1.007	1.101
**hsa-miR-4662a-5p**	0.002	1.298	1.102	1.529
**hsa-miR-6510-3p**	0.076	1.058	0.994	1.127

*miRNAs with OR values less than 1.05 are not shown.

Supplementary Table S6. Genes associated with cervical cancer. Seventy-one genes associated with key processes in HPV-induced carcinogenesis were included to explore their potential regulation by the identified miRNAs and to construct miRNA-gene interaction networks.

Genes	Referencies
BAK1, BAX, BCL2, BRCA1, BRCA2, CADM1, CCND1, CCND2, CCNE1, CDK, CDKN, CDKN1A, CDKN1B, CDKN2A, CDKN2C, CHEK1, CTBP2, CTNNB1, CXCL8, DLG1, E2F, E2F1, E2F7, EGFR, EP300, ERBB2, ERBB3, Fas, FOXO1, FOS, GFBR2, HDAC9, IFNAR1, IGF2R, IGFBP3, IRF3, IRF9, ISGF3, JUN, KDM6B, KRAS, MAML1, MYC, NF1, NFATC1, PIK3CA, PIK3CB, PRKCB, PRKCI, PXN, RABEP1, RABGAP1, RB1, RBL1, RBL2, SCRIB, SIPA1L1, SMAD4, SRC, TERT, TGFBR2, TLR9, TP53, TP53BP1, TP63, UBE3A, UBR4, VEGFA, YY1, p16INK4a, p21, p27.	21,22,23,24
